# Incorporating Yttrium-90 trans-arterial radioembolization (TARE) in the treatment of metastatic pancreatic adenocarcioma: a single center experience

**DOI:** 10.1186/s12885-016-2552-2

**Published:** 2016-07-18

**Authors:** Alexander Y. Kim, Keith Unger, Hongkun Wang, Michael J. Pishvaian

**Affiliations:** Department of Radiology, Division of Interventional Radiology, Medstar Georgetown University Hospital, 3800 Reservoir Rd NW, Washington, DC USA; Department of Radiation Oncology, Medstar Georgetown University Hospital, 3800 Reservoir Rd NW, Washington, DC USA; Department of Biostatistics, Bioinformatics, and Biomathematics, Georgetown University, 3800 Reservoir Rd NW, Washington, DC USA; Department of Medical Oncology, Lombardi Comprehensive Cancer Center, Medstar Georgetown University Hospital, 3800 Reservoir Rd NW, Washington, DC USA

**Keywords:** Pancreatic cancer, Liver metastases, Yttrium-90, Radioembolization, Liver-directed therapy, TARE, SIRT

## Abstract

**Background:**

The purpose of this retrospective study was to evaluate the efficacy of incorporating trans-arterial radioembolization (TARE) with systemic chemotherapy in the treatment of liver-dominant metastatic pancreatic ductal adenocarcinoma, with the aim of destroying liver metastases and improving patient outcomes.

**Methods:**

We retrospectively evaluated 16 patients with liver-dominant metastatic pancreatic ductal adenocarcinoma who underwent TARE between February 2012 and August 2015; 15 of these patients also underwent concurrent systemic chemotherapy. Patient outcomes were assessed using Response Evaluation Criteria In Solid Tumors (RECIST), Version 1.1 and included disease response, median overall survival from the time of diagnosis of metastatic disease, and median overall survival following receipt of TARE. Treatment-related adverse events were assessed using Common Terminology Criteria for Adverse Events (CTCAE), Version 4.03.

**Results:**

The median overall survival from the time of diagnosis of metastatic disease and following receipt of TARE was 22.0 and 12.5 months, respectively. Overall and liver specific disease response were assessed for 13 patients with follow-up imaging available at the time of study (range 2–13 weeks post TARE). Four patients (31 %) demonstrated partial response and five patients (38 %) had stable disease in the liver at follow-up. One patient developed grade 3 elevation of total bilirubin three months post-treatment and another patient developed radiation cholecystitis directly following TARE. No treatment-related grade 4 or 5 toxicities were seen.

**Conclusion:**

TARE can be safely combined with systemic chemotherapy for the treatment of liver-dominant metastatic pancreatic cancer. Patient outcomes following this treatment strategy are promising but prospective evaluations are needed to validate these preliminary findings.

## Background

Pancreatic ductal adenocarcinoma (PDAC) is the fourth leading cause of cancer-related death in the United States [1]. In 2015, there are expected to be 48,960 new cases, and 40,560 deaths from pancreatic adenocarcinoma. The mortality rate of pancreatic cancer is 98 % worldwide [2] and continues to rise [3].

The only potentially curative treatment option for PDAC is surgical resection. However, only 10–20 % of patients are eligible for resection at presentation [2], and most of those will eventually relapse following surgery [4].

Over half of all PDAC patients have metastases at presentation. Recent trial findings indicate improved outcomes for patients with metastatic pancreatic cancer and median overall survival times of 8.5 – 11 months [5, 6]. However, population based studies demonstrate continued poor outcomes in the community, with a median overall survival time of only 4 months [7]. The estimated 5-year survival rate for patients with metastatic disease is only 2.4 %.

Trans-arterial radioembolization (TARE) is a form of liver-directed brachytherapy for treatment of primary and secondary liver cancers [8]. Based on the liver’s dual blood supply, intra-arterial delivery of Yttrium-90 radioactive particles allows for increased uptake of radioactivity into tumor tissue compared with normal liver tissue, which is predominantly supplied by the portal vein.

TARE has been shown to effectively achieve local control and/or improved survival in various tumor types, including hepatocellular carcinoma [9, 10], metastatic colorectal cancer [11, 12], neuroendocrine cancer [13, 14], cholangiocarcinoma [15, 16] and breast cancer [17, 18].

The poor outcomes of currently available therapy for metastatic pancreatic cancer combined with the newly found efficacy of TARE on other types of secondary liver cancers has led to the incorporation of TARE into the treatment arsenal for this patient population. Here we present our single center experience in the use of TARE for management of metastatic pancreatic adenocarcinoma.

## Methods

The retrospective study reported in this paper was approved by the local institutional review board. From February 2012 to August 2015, 16 patients with liver metastases from pancreatic adenocarcinoma were evaluated and treated with TARE (SIR-Spheres, Sirtex Medical, Sydney, Australia) at a single academic institution. All patients were found to have acceptable performance status (ECOG < 2), adequate hepatic reserve (defined as total bilirubin < 3.0 mg/dL and AST/ALT < 5x upper normal limit), and < 50 % of total liver volume replaced with tumor.

Prior to TARE, all patients underwent a complete visceral angiography. In order to reduce the risk of GI ulceration, coil embolization was performed in selected vessels at the discretion of the operating interventional radiologist. With the microcatheter tip at the position of the planned treatment delivery, a dose of technetium-99 m microaggregated albumin (^99m^Tc-MAA) was delivered: 111 MBq and 75 MBq for the right and left lobes, respectively. The patient then underwent a SPECT scan to calculate the percentage of lung shunting, and to detect potential gastrointestinal delivery. The planned treatment dose of resin Yttrium-90 was calculated according to patient body surface area. [8] Dose reduction was performed for high lung shunts as per manufacturer recommendations.

Yttrium-90 radioembolization was performed 1–2 weeks after the mapping angiogram. Delivery of radiation particles to the right and left lobes was performed with the microcatheter positioned in the right or left hepatic artery, respectively. Four patients underwent lobar treatments in separate sessions spaced 14–56 days apart. Six patients underwent single session whole liver treatments in order to minimize their time away from systemic therapy. In order to minimize potential risks of gastrointestinal ulceration in patients undergoing whole liver treatments, the radiation particles were delivered in a lobar fashion using a “split dose” strategy. Following treatment, all patients were observed for a 4–6 h period and then discharged.

Fifteen patients were being treated with concurrent systemic therapy at the time of TARE. Six patients were receiving a 5-FU-based regimen; eight were receiving concurrent gemcitabine-based therapy, and one patient was receiving Abraxane only. Information regarding the timing of systemic chemotherapy infusions was available for 13 patients only. Systemic therapy was stopped on average 18 days (range 8–50 days) prior to TARE, and was resumed on average 22 days (range 6–46 days) after TARE.

Follow-up imaging assessment was carried out two to six months after the final TARE treatment session. Local and overall disease response was assessed using RECIST (v 1.1) guidelines by comparison of each follow up imaging with the baseline study. Pre and post treatment laboratory values were retrospectively evaluated to assess for adverse events as defined by CTCAE v 4.3.

Descriptive statistics were used to summarize patients’ characteristics as well as their treatment parameters. Kaplan-Meier methodology was used to analyze patients’ survival data. The median overall survival time from diagnosis of liver metastasis to time of analysis was estimated with its 95 % confidence interval when feasible. SAS software version 9.3 (SAS Inc., Cary NC) was used in the data analysis.

## Results

Eleven male and five female patients were treated for liver-dominant metastatic PDAC with Yttrium-90 resin microspheres directed to the liver. All patients were treated with at least one line of prior or concurrent chemotherapy at the time of TARE (range 1–3). One patient had undergone a pancreaticoduodenectomy prior to developing metastatic disease. Five patients underwent SBRT to the primary pancreatic lesion, and one patient had prior chemoembolization for the treatment of liver disease.

A summary of baseline patient characteristics is outlined in Table 1.

**Table 1:** Baseline patient characteristics

Variable	Patients
Total	16
Male	11
Female	5
Age	63 (range 50 – 73)
Performance status	
0	8
1	8
Location of primary lesion	
Head/Neck	8
Body/Tail	8
Prior (concurrent) chemotherapy	
Gemcitabine based	8
5-FU based	5
Both	3
Prior treatment	
Pancreaticoduodenectomy	1
SBRT	5
DEBIRI-TACE	1
Other	2
Median Total Bilirubin	0.6 (range 0.2 – 2.6)
Median Albumin	3.5 (range 2.2 – 4.4)
Median CA 19-9	15,836 (range 2 – 76587)
Hepatic tumor burden	
<25 %	13
25-50 %	3
Extra-hepatic disease	
Lymph node	9
Lung	3
Peritoneal	3
Other	1

Ten patients underwent whole liver treatment and six underwent single lobe treatment. Six of the 10 patients undergoing whole liver treatment were treated in a single session with the lobes treated in a split fashion. There was an average interval of 28 days (range: 14–57 days) in between treatments for the four patients undergoing lobar treatments. The average doses of Yttrium-90 delivered to the right and left lobes of the liver were 46.32 Gy (30.24 – 65.10) and 54.44 Gy (32.31 – 86.29), respectively (Table 2).

**Table 2:** Treatment parameters

Treatment	
Single lobe	6
Both lobes	10
Single session	6
Two sessions	4
Prescribed activity	
Right lobe	1.15 GBq (range 0.73 – 1.64)
Left lobe	0.74 GBq (range 0.40 – 1.04)
Administered activity	
Right lobe	1.00 GBq (range 0.55 – 1.74)
Left lobe	0.64 GBq (range 0.35 – 0.91)
Lobar dose	
Right lobe	46 Gy (30 – 65)
Left lobe	54 Gy (32 – 86)

**Fig. 1:**
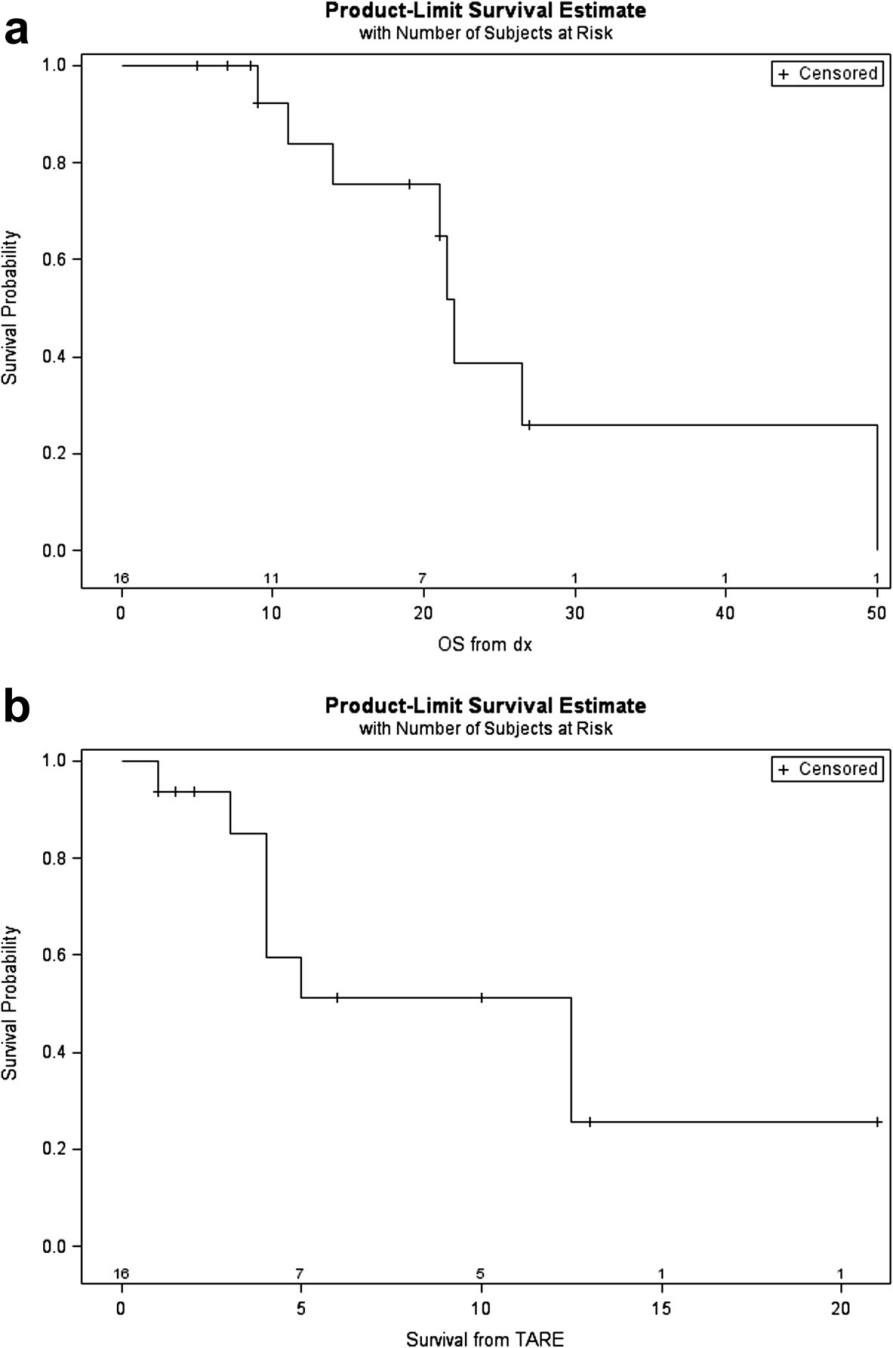
Median overall survival (**a**) Kaplan-Meier curve demonstrating median overall survival from diagnosis of metastatic pancreatic ductal adenocarcinoma (**b**) Kaplan-Meier curve demonstrating median overall survival from TARE

Eight of the 16 patients were alive at the time of this retrospective analysis. The median overall survival from the time of diagnosis of metastatic disease to time of this retrospective analysis was 22 months (range: 11 – 50) (Fig. 1a). The median time of survival from TARE is 12.5 months (range: 4 – not reached,) (Fig. 1b). The estimated survival rate from diagnosis at 6 months, 1 year, 2 years, and 3 years is 100 %, 84 %, 39 %, and 26 %.

The single patient who had local disease and underwent a pancreaticoduodenectomy with subsequent development of metastatic disease had an overall survival of 50 months from diagnosis of metastases, however survived only 4 months from initial TARE. Two of the five patients who were treated with SBRT for their primary lesion are still alive at the time of assessment. The median overall survival and survival from TARE are 18 months (range 11 months – not reached) and 9.4 months (range 3 months – not reached), respectively.

**Fig. 2:**
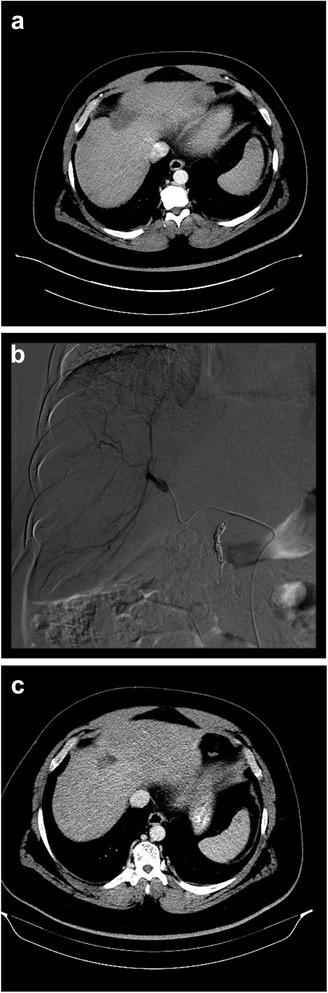
Pre, intra, and post TARE imaging (**a**) Pre-procedural CT demonstrating a segment 4 metastatic pancreatic adenocarcinoma measuring 4.4 x 2.2 cm (**b**) Hepatic angiogram demonstrating enhancing segment 4 mass with aberrant arterial vasculature (**c**) 3 month post procedure CT demonstrating PR to TARE with lesion now measuring 2.8 x 1.8 cm

Follow up imaging was available for 13 patients (Fig. 2). Local partial response (PR), stable disease (SD), and disease progression (PD) based on RECIST criteria were seen in four (31 %), five (38 %) and four (31 %) patients, respectively. The average local and systemic progression-free survival (PFS) was 4.9 and 3.4 months, respectively (Table 3).

**Table 3:** Local and systemic disease response

Patient number	Best local response	Time from treatment (months)	Best systemic response	Time from treatment (months)
1	SD	1	SD	1
2	PD	3	PD	3
3	PR	3	PR	3
4	PR	5	PR	5
5	N/A		N/A	
6	SD	1.5	SD	1.5
7	SD	10	SD	6
8	PD	3	PD	3
9	SD	8	SD	4
10	PD	3	PD	3
11	SD	6	SD	3
12	PR	2	PD	2
13	PD	2	PD	2
14	PR	2	PR	2
15	N/A		N/A	
16	N/A		N/A	

Liver function tests (AST, ALT, alkaline phosphatase, and total bilirubin) were available at one month post-TARE for 15 patients, and three months post TARE for 12 patients. No treatment related grade 3 or higher hepatic dysfunction was demonstrated at 1 month, although one patient developed grade 3 elevation of total bilirubin at three months. No grade 4 or 5 elevation of any liver function tests was seen at three months. One patient died 40 days following treatment, presumably due to disease progression. Another patient developed transient radiation cholecystitis and hepatitis following therapy but his liver enzyme levels returned to baseline after one month.

## Discussion

Trans-arterial radioembolization has been used with increasing frequency to treat a variety of liver dominant secondary cancers. Given the limited efficacy of systemic therapy alone for patients with metastatic pancreatic adenocarcinoma, we have incorporated TARE with the hope of better managing the outcome of patients.

There is currently limited published experience regarding the incorporation of liver-directed therapy for management of metastatic PDAC. Azizi and colleagues (2011) published their findings on the role of repetitive trans-arterial chemoembolization (TACE) for the management of metastatic pancreatic adenocarcinoma [19]. Thirty-two patients underwent conventional TACE (cTACE) using a triple chemotherapeutic regimen of mitomycin C, cisplatin, and gemcitabine administered as an emulsification with Lipiodol followed by embolization with 200–450 micrometer microspheres. Based on RECIST criteria, the disease control rate in this treated cohort was 81 % (PR 9 %, SD 72 %), and the median overall survival was 16 months.

There are two published retrospective studies assessing the efficacy of TARE in the management of a PDAC patient population. First, Cao et al. (2010) retrospectively evaluated seven patients with liver metastases from pancreatic adenocarcinoma [20]. Follow-up imaging was available for five patients. Two patients demonstrated PR, one had SD, and two had PD following TARE. Survival data was not presented but it was noted that one patient was still alive 15 months after treatment. No adverse events were noted for any of the treated patients.

More recently, Michl et al. (2014) published their experience with TARE as a treatment for 19 patients with metastatic pancreatic adenocarcinoma [21]. Twelve patients had liver-dominant metastatic disease, and seven had liver-only disease at the time of TARE. The majority (84 %) had received prior palliative chemotherapy. At first follow-up, 64 % of patients demonstrated anti-tumor response in the liver, and 36 % demonstrated progression of disease. Following TARE, median overall survival from time of initial diagnosis was 9.0 months in the patients with liver-dominant metastatic disease, and 18.9 months in patients that had liver-only disease. One patient experienced treatment-related gastric ulceration requiring gastrectomy. Two patients developed hepatic abscesses following treatment, and two others developed signs of radioembolization induced liver dysfunction (REILD).

As opposed to the studies by Michl and Cao, we utilized a novel strategy of incorporating TARE with systemic chemotherapy, as opposed to using TARE as a salvage therapy. A short break of systemic therapy was added during time of TARE to reduce risks from radiosensitization and/or patient fatigue with an average of 40 days between cycles. Despite concurrent treatment of patients with systemic chemotherapy—eight of who received gemcitabine-based therapy, we encountered minimal treatment-related hepatotoxicity with only one patient developed grade 3 elevation of total bilirubin three months post-TARE.

The retrospective nature of our study means that it has inherent weaknesses. The sample size is small – data is reported from only 16 treated patients – and lacks a control group for comparison. Furthermore, given the selection criteria for treatment, patients with rapidly progressing disease were excluded from the study, leading to potential selection bias. The findings from our study, however, with a median overall survival of 22.0 months from diagnosis and 12.5 months from TARE, compares favorably with the established overall median survival of 8.5–11 months demonstrated with systemic chemotherapy alone for patients with metastatic pancreatic cancer [5, 6]. The apparent safety and improved efficacy of our treatment support the strategy of incorporating TARE with concurrent chemotherapy in the treatment of patients with liver-dominant metastatic pancreatic ductal adenocarcinoma. Further prospective evaluation of the efficacy of this treatment strategy is warranted.

## Conclusions

Incorporation of Yttrium-90 radioembolization to standard chemotherapy appears to be safe may improve patient outcomes. Prospective evaluations are warranted to validate our findings.

## Abbreviations

CTCAE, common terminology criteria for adverse events; PD, disease progression; PDAC, pancreatic ductal adenocarcinoma; PFS, progression-free survival; PR, partial response; RECIST, response evaluation criteria in solid tumors; SD, stable disease; TACE, trans-arterial chemoembolization; TARE: trans-arterial radioembolization
